# A Few Cases in Surgical Practice

**Published:** 1891-05

**Authors:** T. Dwight Stow

**Affiliations:** Mexico, N. Y.; Bureau of Surgery, I. H. A.


### A FEW CASES IN SURGICAL PRACTICE.

T. Dwight Stow, M. D., Mexico, N. Y.

(Bureau of Surgery, I. H. A.)

This report of surgical cases is not made because of any particular merit in the operative measures, but as showing the merit of Homœopathy, in meeting whatever demand is made upon it for the welfare and general comfort of such cases, and to further show how unnecessary are the usual routine exhibits of the old school.

Mrs. D. S., a lady forty-eight years of age, one year and six months ago noticed a sensitive tumor in the axillary border of right breast.

The tumor was subject to paroxysms of heat and sharp lancinating pain ; was firm, hard, nodulated, and steadily increased in size until it involved nearly one-half the breast. At its summit it inflamed and ulcerated, and occasionally bled. She was not strictly cachectic, but began to have not a little rise of temperature, some thirst, and considerable alarm when she applied to me for an operation. Putting her under treatment for a month or more we set a day for the operation and removed the entire breast, December 12th, 1889. She made a nice recovery ; the wound healed by the first intention, and she had but four prescriptions, two of them Sac-lac.

The first prescription given was Bell.$^{500}$. It essentially modified the inflammatory symptoms, the soreness, sensitiveness to touch and the swelling, fever, and redness of face. Pulsatilla$^{200}$ was given after the operation for the relief of dyspnœa, worse in a warm room ; and nausea at night sometimes aggravated by the *odor* of the ejecta.

Fracture of right tibia, followed by extensive ecchymosis, blistering and exfoliation of the epidermis.

David D., a mechanic, sixty years of age; a man addicted to the use of the ardent, in an intoxicated condition fell from the steps of his shop upon the sidewalk, breaking his right tibia in its lower third.

The fracture was long and oblique, the lower point of upper fragment nearly penetrating the skin on the inner face of the tibia. As the patient was very garrulous and uneasy, we anæsthetized him, reduced the fracture, confining the limb in an anterior and posterior Ahl's porous felt splint. Three days after the fracture, I was obliged to re-dress the whole fractured limb, on account of great swelling, erysipelatous inflammation, and the formation of many large bullæ, that covered at least three-fourths of the front and sides of the limb. Ulceration had taken place over the lower line of fracture, so that it was necessary to adjust the limb in a fracture box, packing it with fresh, sifted and baked pine saw-dust. He complained of burning heat; soreness, aching in the limb, and instinctively put out his hand to keep people away from his limb. Arnica[30] made him very easy, and after the second night he slept, on the average, six hours, getting, also, naps by day. His limb is now doing well; is of good shape and length; there is slight œdema of the foot, and he is fully convalescent. The limb was fractured May 6th, and now, June 10th, he is wearing a starch bandage and getting about on crutches.

He has had no whiskey, no alcoholic stimulant, no Morphine, no Chloral, no anodyne, no physic; but is doing nicely in all visible respects.

In closing, let me speak in praise of nice, clean baked, browned or slightly charred pine saw-dust in the treatment of fractures with suppurative, serous, or sanguineous discharges, erysipelatous inflammation, etc.

### EPITHELIOMA.

About the middle of November, 1889, an old gentleman, a farmer by occupation, came to get a prescription for a sore on his lower lip, a little to the left of the raphe. The tumor was quite hard, well-defined, as large as an ordinary chestnut; had on its surface a dirty gray, slimy mucus, which, when wiped off, revealed a reddened surface. Under a magnifying lens it had the characteristics of the columnar or cylindrical variety of cell. He complained of great soreness, burning; some sharp cutting pain in the tumor, worse in the wind or out-of-doors; better near the stove; better when covered with adhesive plaster or lint. He was also worse before, or just after midnight, and he was much inclined to be chilly. I put up a few powders of Ars-alb.$^{300}$, one prescription. The Ars. mitigated the subjective symptoms above enumerated, and on the 15th of February, assisted by Dr. Bennett, of Mexico, N. Y., I excised the tumor by an incision, cutting away about one-half the lip, the wound thus formed being an equilateral triangle. The edges were approximated by transfixion with silver pins, and figure-of-eight ligature. Union was perfect on the fourth day, and he made a fine recovery. He called on me, Friday, June 6th, a well man visibly. There is no scar visible; only a preternatural tension and attenuation of the labia.

### A Queer Case!

Wednesday, May 28th, 1890, a physician of our town of Mexico, N. Y., called to take me to see a patient of his that, to use his language, "puzzled him." The patient, a young man of twenty years, unmarried, was very sick, having much fever, thirst, restlessness; temperature 104 3-5°; frequent pulse; tongue coated white, dry and red in the centre, red edges, a dry red triangular tip; lips dry, with a tendency to scale. The penis and its gland were enormously swollen and inflamed, and commencing on the dorsum, behind the corona, was a sloughing phagedenic ulcer that rapidly spread from a pimple on Friday, May 23d, to a foul ulcer, destroying all tissue down to the corpus spongiosum, bounded by the frænum preputium below. Para-phimosis was present, and a large abscess was formed along the dorsum of the organ. The lower portion of the prepuce, each side of the frænum was very œdematous, and the whole organ very sore, and painful to touch or movement. During the night and morning there was frequent and profuse hemorrhage from the ulcer. We controlled hemorrhage by sub-integument transfixion of the dorsalis penis artery. Some three months prior to this, the patient contracted gonorrhœa, which was treated in the usual way by old-school methods.

Some three days prior to the acute attack, he got warm and sweaty while working on the track of the Rome, Watertown, & Ogdensburg R. R., and was caught in a heavy but warm shower, and wet through. The attack mentioned was ushered in by a shaking chill, aching of bones, backache, thirst, etc. All in all, he was in a pitiable condition.

He stoutly denied having any unclean connection whatever, since he had gonorrhœa, but the edges of the ulcer were raised and hard, and of a suspicious character. I should state that his physician applied Carbolic acid to the ulcer two or three times. For two more days the case was alarming. We slit up the prepuce to relieve the constriction and give vent to the rapidly-accumulating matter, thoroughly irrigated the ulcer with hot water and a weak solution of Lloyd's asepsin ; covered the parts with Lister protection and plain absorbent cotton. Internally we gave Rhus-tox.[30] He began to improve at once, and on June 1st, he was out of danger, the sloughing and foul, cadaverous smelling purulent discharge having ceased, the ulcer looking clean and paler. The gland, now almost detached from the penis, we kept in position by means of a rubber stem inserted in the urethra to steady it, and by adhesion strips confined to the dorsum and sides of the penis. His fever, high temperature, frequent pulse, dry tongue, and thirst disappeared June 2d, and he is now convalescent. The gland has united to the body of the organ, and is covered daily with fresh protective and cotton.

I neglected to state in the proper place that during the febrile stage there was ischuria requiring the use of the catheter, also painful priapism.

Query :—Was this a phagedenic chancre, or was the frightful ulceration due to the Carbolic acid ?

If it were a chancre, the rapid recovery was simply amazing.

If the ulcer were in the main, syphilitic, and the patient innocent, how did the patient become victimized?

If the phagedenic ulcer were produced by the Carbolic acid, what shall be said of such treatment?

